# Contemporary trends in anaphylaxis burden and healthcare utilisation in Western Australia: A linked data study

**DOI:** 10.1016/j.waojou.2023.100818

**Published:** 2023-09-27

**Authors:** Samantha L. Stiles, Frank M. Sanfilippo, Richard Loh, Maria Said, Rhonda M. Clifford, Sandra M. Salter

**Affiliations:** a School of Allied Health, The University of Western Australia, Perth, Australia; b School of Population and Global Health, The University of Western Australia, Perth, Australia; c Medical School, The University of Western Australia, Perth, Australia; d Perth Children's Hospital, Perth, Australia; e Allergy & Anaphylaxis Australia, Sydney, Australia; f National Allergy Strategy, Sydney, Australia; g Australasian Society of Clinical Immunology and Allergy (ASCIA), Sydney, Australia

**Keywords:** Anaphylaxis, Epidemiology, Linked data, Trends, Australia

## Abstract

**Background:**

Anaphylaxis is a growing public health problem in Australia. To determine the extent of the problem, we linked multiple health datasets to examine temporal trends in anaphylaxis events across the health system in Western Australia (WA).

**Methods:**

We identified an anaphylaxis cohort from 1980 to 2020 using linked datasets from ambulance, emergency departments, hospital inpatients and deaths. Age-standardised anaphylaxis event rates were calculated from 2010 to 2020. Dataset-specific rates for anaphylaxis were also examined, to show differences in health care utilisation. Annual percent change in rates (2010–2019) were estimated using age-adjusted Poisson regression models.

**Results:**

A total of 19 140 individuals (mean age 31 years; 51% female) experienced 24 239 anaphylaxis events between 2010 and 2020. From 2010 to 2019, the average annual percent increase (95% CI) in rates was 5.3% (4.8–5.8%), from 70.3 to 113.9, with rates reducing to 76.5/100 000 population in 2020. Adolescents and young adults aged 5–14 years and 15–24 years had the greatest increase of 6.9% (5.6–8.1%) and 6.8% (5.6–8.0) respectively, with those over 25 years increasing by approximately 5% per year and children 1–4 years showing the lowest annual increase of 2.6% (1.1–4.2%). The highest absolute rates were seen in under 1 year (269.7/100 000; 2019). There has been an acceleration of trends from 2015 to 2019, underpinned by large increases in 15–24 and 25–34 years. All databases, show similar increasing trends, with ambulance attendance (33.7 per 100 000), emergency presentation (89.8 per 100 000) and hospital admissions (46.2 per 100 000), for anaphylaxis highest in 2019. However, whilst ambulance and emergency presentations have grown by 8.9% (95%CI 7.9–9.8%) and 6.6% per year (95%CI 6.0–7.2%), respectively, hospitalisations appear to be steadying with only a 0.9% (95%CI 0.2–1.6%) yearly rise.

**Conclusion:**

Rates of anaphylaxis continue to increase, with WA having higher rates than previous estimates for Australia. Whilst rates are still high in infants, lower trends in children compared to older ages may indicate better prevention of allergy. Results show more people experiencing anaphylaxis now receive care in emergency and ambulance, rather than hospital. Further exploration of the patient care journey through prehospital and inpatient care is required, to understand the changing health demands of people who experience anaphylaxis.

## Introduction

The public health impact of anaphylaxis, characterised as a rapid and potentially life-threatening allergic reaction, is increasing.^1^ The true burden of anaphylaxis is difficult to determine due to inconsistent reporting of events and because episodes commonly occur in the community outside of a hospital setting.^2^ In the absence of structured reporting, the analysis of routine health data collections can provide valuable insights into the current burden and healthcare utilisation for people experiencing anaphylaxis. Previous studies have mostly considered anaphylaxis in single clinical settings, most commonly utilising emergency department or hospital admissions data.^2^ These likely underestimate the true burden of anaphylaxis, capturing only the most severe cases, with only a small proportion being admitted.^2^,^3^ Australia has a universal healthcare system and anaphylaxis is managed across several settings. As anaphylaxis is a medical emergency, the inclusion of ambulance and emergency presentations would provide better case capture. Particularly as Australian guidelines recommend that a person experiencing anaphylaxis should call an ambulance and be medically observed for a minimum of 4-h after their last dose of adrenaline.^4^

Linking multiple health datasets that captures where patients go to receive care allows more comprehensive person-based epidemiological estimates to be obtained. The “*blinded for review*” Study, which reported on anaphylaxis using linked data in Western Australia (WA) from 2002 to 2013, has provided evidence that rates at that time were substantially higher than those previously reported, both in Australia and worldwide.^5^ Considering, more recent studies have indicated that rates of anaphylaxis have continued to rise, particularly food and medication related reactions, we expect to see a further increase in rates in WA.^6^, ^7^, ^8^, ^9^, ^10^, ^11^ Recent data on food-related anaphylaxis hospitalisations in Australia have indicated a slowing of the rates in young children in recent years.^6^ This will be important to monitor to highlight any shifts in the demographic profile of people at risk of anaphylaxis.

Our study, the Anaphylaxis Epidemiology and Characteristics in Western Australia (ACE-WA) study uses linked ambulance, emergency department, hospital inpatient and death datasets to identify a large anaphylaxis cohort in WA from 1980 to 2020. This study provided an update to epidemiological estimates from 2010 to 2020. The primary aim of this study was to identify contemporary trends in anaphylaxis event rates, stratified by age and gender. The secondary aim was to examine trends in health service utilisation and distribution of events by cause.

## Methods

### Study design

The ACE-WA study is a retrospective linked data study using whole population administrative health data from multiple sources.

### Data sources and data linkage

Data were obtained from the Western Australian Data Linkage System (WADLS), which creates and maintains person-specific linkages within and between health and non-health datasets.^12^ This study established a file of linked records based on four datasets relevant to anaphylaxis. These are: St John Ambulance (SJA) WA; the Emergency Department Data Collection; Hospital Morbidity Data Collection; and WA Deaths Register. These datasets will be hereafter called "ambulance", "emergency", "inpatient", and "deaths", respectively.

The hospital dataset provides coverage of all inpatient hospitalisations in WA across the public and private systems. The emergency dataset includes attendance at any public and private hospital emergency department in WA. This includes admissions to the emergency observation ward, particularly relevant to the treatment of anaphylaxis. SJA is an independent organisation, contracted by WA Health to provide state-wide ambulance service and is the sole provider in WA. Full state coverage of the ambulance (electronic records from 2011), inpatient and mortality datasets are available from 1980 onwards, and emergency data from 2002 onwards.

### Study population

The population for this study includes all individuals in WA with a record for anaphylaxis in 1 or more of the linked datasets from 1980 to 2020. A subset of this data, from 2010 to 2020, is analysed in the current paper, referred to as the analysis cohort. This time-period was selected to provide a contemporary view of anaphylaxis burden and health service demand and to update previous WA anaphylaxis rates based on similar methods.^5^ In light of changing guidelines and awareness of anaphylaxis since 2010, this is important to inform current policies and practices for the reporting and treatment of anaphylaxis. At the time of extraction, data were incomplete for the year 2020 (data available from 1 January to October 31, 2020). Therefore, anaphylaxis event frequencies were extrapolated by adding the average monthly number of events (by gender, age group, and trigger) for 2020 to the total year count.

**Fig. 1:**
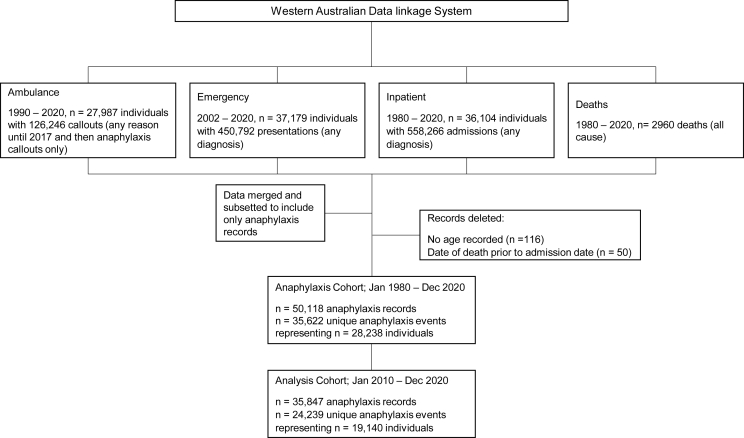
Data flowchart for anaphylaxis cohort.

Anaphylaxis records were identified using ICD-10-AM (International Statistical Classification of Diseases and Related Health Problems 10th revision Australian Modification), emergency symptom code and ambulance problem code specific to anaphylaxis (see Supplementary material, Table S1 for codes). Linked records (all anaphylaxis-coded records for each individual in the anaphylaxis cohort, plus all other non-anaphylaxis records for those individuals) were extracted for available time periods, for ambulance (n = 27 987), emergency (n = 37 179), inpatient (n = 36 104) and deaths (n = 2960). After data were cleaned and merged, the anaphylaxis cohort included 35 622 unique anaphylaxis events (using the definition for events below) for 28 238 individuals. The analysis cohort includes 24 239 events representing 19 140 individuals from January 2010 to December 2020 (Fig. 1).

### Measures of anaphylaxis occurrence

As people experiencing anaphylaxis may receive care across multiple health settings during a single reaction, distinct "events" where identified to avoid over counting. A series of anaphylaxis records from multiple datasets (or the same dataset), were considered part of a single event if they occurred within 24 h of the previous anaphylaxis record.

To examine trends in anaphylaxis rates, we considered 2 measures:

1. Event-based rates, in which anaphylaxis records pertaining to the same event were counted once. This was used to calculate the burden of anaphylaxis and was the main measure reported. Cause-specific rates (the number of events for each trigger), was calculated to show distribution of events by cause.
2. Record-based rates, in which every record for anaphylaxis was counted by dataset. This was done to allow comparisons with previous epidemiological studies that only utilise unlinked single administrative datasets and to show dataset specific health utilisation.

### Allocation of triggers

Anaphylaxis events were labelled as "food", "medication" or "unspecified" based on recorded ICD-10-AM codes. All ambulance records were labelled unspecified, as no cause-specific codes are available. Serum was excluded due to low numbers (303 records from 2010 to 2020).

Where events include more than one linked record (and therefore more than one anaphylaxis code), a trigger was allocated to the event according to the following rules: 1) Specificity of code (ie, "food" or "medication" as opposed to "unspecified") within an event series; and 2) Hierarchy of diagnosis setting (Inpatient > Emergency > Ambulance) within an event series.

### Demographic variables

Key demographics, including date of birth, gender, and Socio-Economic Indexes for Areas (SEIFA) were derived and harmonized using information from all linked datasets. For date of birth and gender, the mode of all observed values for a person was chosen within and across the available data collections.

The SEIFA used was the Index of Relative Socio-Economic Advantage and Disadvantage (IRSAD), which is a general measure of economic and social conditions of people and households within an area.^13^ The IRSAD scores from the 2016 Australian census were used, grouped into quintiles for analysis, with quintile 1 representing the most disadvantaged area and quintile 5 representing the least disadvantaged area.

### Statistical analysis

Annual age-standardised rates (<1, 1–4, 5–14, 15–24, 25–34, 35–64, and 65 years and over) were calculated by the direct method using 5-year age groups, and the 2016 Australian standard population was used for age standardisation. Numerators for either event-based or record-based rates were the annual number of events or records for anaphylaxis respectively. Denominators were annual WA population numbers. For cause-specific event rates the numerator was the annual number of events for each allocated trigger (based on above rules). Age-adjusted trends for rates were estimated using Poisson regression models, with 5-year age group and year (continuous) in the models. Analyses for event rates and trends were calculated separately for men and women and for genders combined.

To separate early and contemporary changes, trends were calculated for the whole study period and separately for 2010–2014 and 2015–2019. This allows investigation of any shift in drivers of trends between genders and ages and in relation to cause. Preliminary analysis indicated that rates dropped significantly in 2020. This is likely due to the impact of lockdowns associated with the COVID-19 pandemic during this time as well as having incomplete data. Therefore, whilst rates were calculated for this year, data for 2020 was not included in trends analysis to avoid skewing of the results (see Supplementary Material Table S2 to see trends from 2010 to 2020).

## Results

### Cohort characteristics

From 2010 to 2020, a total of 19 140 individuals experienced 24 239 anaphylaxis events (Table 1). Slightly over half of the events (51.0%) occurred in females and the mean age was 31 years (min age: 1 month; max age: 102 years), which remained relatively stable over the study period. Most people experiencing anaphylaxis are those living in areas of greater socioeconomic advantage (quintile 5, 49.4%). Most events were coded as unspecified (16 267; 67.1%), followed by food (4393; 18.1%) and medication, (3579; 14.8%). The majority (79.2%) of individuals experienced 1 anaphylaxis event, with 17% experiencing 2 to 4 events (range 1–147). There were 13 recorded anaphylaxis deaths, with all but 1 occurring in adults (over 30 years). None had an ICD code for anaphylaxis as the primary cause (See Supplementary Material, Table S3 for primary cause of death).

**Table 1:** Characteristics of anaphylaxis events in Western Australia from linked health data, in the anaphylaxis cohort, 2010–2020

	Anaphylaxis events, n (%)				
	Total 2010–2020	2010	2015	2019	2020^a^
Total number of people	19,140	1511	1783	2222	1349
Total number of events	24,239	1638	2268	3038	2080
Mean age at event, y(SD)	31.1 (21.6)	31.2 (21.7)	30.3 (21.3)	31.3 (21.8)	30.4 (21.6)
Gender at event					
Female	12 367 (51.0)	800 (48.8)	1117 (49.3)	1639 (53.9)	1092 (52.5)
Age group,y					
<1	697 (2.9)	48 (2.9)	68 (3.0)	90 (3.0)	60 (2.9)
1–4	2166 (8.9)	170 (10.4)	189 (8.3)	252 (8.3)	199 (9.6)
5–14	3848 (15.9)	207 (12.6)	388 (17.1)	485 (16.0)	338 (16.3)
15–24	3986 (16.4)	278 (17.0)	379 (16.7)	520 (17.1)	361 (17.4)
25–64	3531 (14.6)	237 (14.5)	317 (14.0)	454 (15.0)	282 (13.6)
35–64	7972 (32.9)	575 (35.1)	750 (33.1)	951 (31.3)	677 (32.6)
65+	2038 (8.4)	123 (7.5)	177 (7.8)	286 (9.4)	162 (7.8)
Event trigger^b^					
Food	4393 (18.1)	321 (19.6)	377 (16.6)	568 (18.7)	252 (12.1)
Medication	3579 (14.8)	283 (17.3)	353 (15.6)	383 (12.6)	173 (8.3)
Unspecified	16 267 (67.1)	1034 (63.1)	1538 (67.8)	2087 (68.7)	1655 (79.6)
Health service^c^					
Total number of records	35847	2442	3293	4544	3048
Total ambulance callout	7029 (19.6)	380 (15.6)	656 (19.9)	896 (19.7)	900 (29.5)
Total emergency presentations	17 976 (50.1)	1187 (48.6)	1619 (49.2)	2404 (52.9)	1636 (53.7)
Total inpatient hospitalisations	10 842 (30.2)	875 (35.8)	1018 (30.9)	1244 (27.4)	512 (16.8)
Deaths					
All-cause deaths	641	12	82	101	92
Anaphylaxis deaths	13	2	0	0	0
Number (%) of people experiencing multiple anaphylaxis events					
2 events	2830 (11.8)	76 (4.7)	127 (5.6)	191 (6.3)	117 (6.8)
3 events	852 (3.6)	16 (1.0)	21 (0.9)	46 (1.5)	20 (1.2)
4 events	379 (1.6%)	5 (0.3)	7 (0.3)	19 (0.6)	4 (0.2)
SEIFA quintile^d^					
1	227 (1.0)	7 (0.4)	28 (1.3)	32 (1.1)	19 (0.9)
2	839 (3.7)	54 (3.4)	73 (3.3)	115 (3.9)	77 (3.8)
3	1956 (8.5)	101 (6.4)	184 (8.4)	297 (10.2)	192 (9.6)
4	8627 (37.5)	594 (37.7)	823 (37.7)	1115 (38.1)	755 (37.7)
5	11 350 (49.4)	821 (52.1)	1076 (19.3)	1367 (46.7)	962 (48.0)

a Counts for 2020 was extrapolated from data available from Jan–Oct 2020.

b Represents "allocated" triggers for events.

c Record-based counts (the same anaphylaxis "event" may be represented across more than one dataset).

d Denominator is 34,026. There were 1119 records missing SEIFA values.

### Average annual total anaphylaxis event rates

**Fig. 2:**
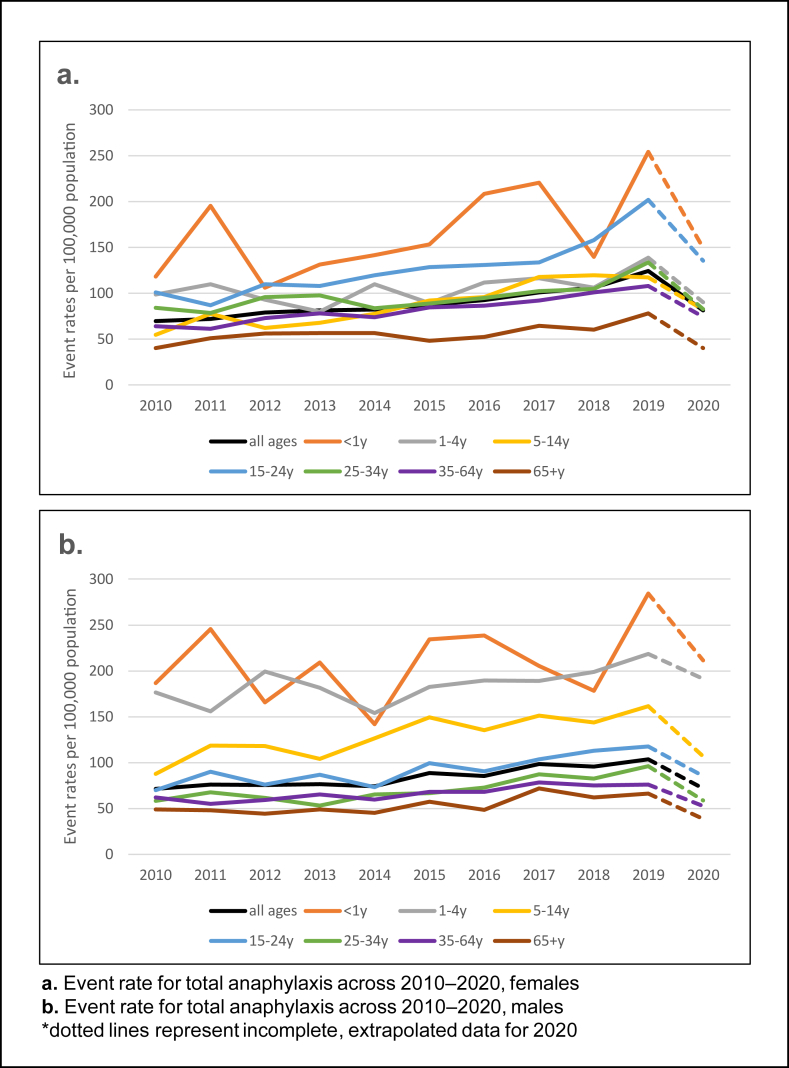
Event rate for total anaphylaxis, 2010–2020.

Age-standardised anaphylaxis event rates increased by 1.6-fold, from 70.3 per 100 000 population in 2010 to 113.9 in 2019, followed by a 1.5-fold reduction to 76.5 per 100 000 population in 2020 (Table 2; Fig. 2). In 2019, rates were higher in females than males (124.2 and 103.7 per 100 000, respectively). Male children under 15 years and females older than 15 years had higher rates. Overall rates peaked in infants under 1 year (269.7 per 100 000; 2019) and reduced with age.

**Table 2:** Age-standardised event rates and trends for total anaphylaxis, 2010–2020

Age-standardised annual event rates per 100,000 population					Average annual % change (95% CI)^a^		
Age (years)	2010	2015	2019	2020	2010–2019	2010–2014	2015–2019
**All persons**							
all ages	70.3	88.1	113.9	76.5	5.3 (4.8–5.8)	2.7 (1.2–4.2)	6.6 (5.3–7.9)
<1y	153.6	195.0	269.7	181.4	4.1 (1.2–7.0)	−4.4 (−12.1 to 4.0)	4.0 (−3.3 to 11.8)
1-4y	138.2	137.0	179.9	142.5	2.6 (1.1–4.2)	−1.0 (−5.5 to 3.7)	5.9 (1.5–10.4)
5–14y	71.7	121.4	140.1	95.0	6.9 (5.6–8.1)	5.7 (1.8–9.8)	4.3 (1.2–7.4)
15–24y	84.8	113.6	158.4	109.7	6.8 (5.6–8.0)	3.4 (−0.2 to 7.1)	9.5 (6.2–12.9)
25–34y	70.8	77.7	114.8	70.8	5.0 (3.7–6.3)	1.0 (−2.7 to 4.9)	9.4 (5.9–13.0)
35–64y	63.0	76.3	91.9	63.5	5.0 (4.1–5.8)	3.3 (0.7–5.9)	5.1 (2.9–7.4)
65 + y	44.2	52.4	71.9	39.2	4.6 (2.9–6.3)	3.4 (−1.7 to 8.8)	8.5 (4.0–13.1)
**Males**							
all ages	71.3	88.6	103.7	71.8	4.3 (3.6–5.0)	0.9 (−1.2 to 3.0)	4.3 (2.5–6.1)
<1y	186.9	234.5	284.5	211.6	2.4 (−1.3 to 6.2)	−6.5 (−16.2 to 4.4)	1.6 (−7.8 to 12.1)
1-4y	176.2	182.5	218.4	191.5	2.3 (0.3–4.3)	−1.2 (−6.8 to 4.7)	4.2 (−1.1 to 9.9)
5–14y	87.9	149.2	161.6	106.8	5.4 (3.8–7.0)	5.7 (0.8–10.8)	2.3 (−1.6 to 6.4)
15–24y	69.8	99.5	117.6	85.4	5.2 (3.4–7.1)	0.5 (−4.7 to 6.0)	5.8 (1.0–10.9)
25–34y	58.1	66.9	96.0	58.9	5.7 (3.7–7.7)	−0.1 (−5.7 to 5.9)	8.6 (3.5–14.0)
35–64y	62.2	68.2	76.0	52.7	3.5 (2.3–4.8)	0.8 (−2.8 to 4.6)	3.1 (−0.2 to 6.5)
65 + y	48.8	57.1	66.2	38.6	4.5 (2.0–7.0)	−1.1 (−8.3 to 6.7)	5.2 (−1.0 to 11.8)
**Females**							
all ages	69.5	87.6	124.2	81.2	6.2 (5.5–6.9)	4.5 (2.4–6.7)	8.8 (6.9–10.7)
<1y	118.4	153.2	254.0	149.3	6.3 (1.9–10.9)	−1.2 (−13.4 to 12.6)	7.0 (−4.1 to 19.3)
1-4y	98.6	89.4	138.8	90.3	3.2 (0.5–5.9)	−0.7 (−8.2 to 7.5)	8.7 (1.2–16.7)
5–14y	54.8	92.2	117.5	82.5	9.1 (7.1–11.2)	5.7 (−0.6 to 12.5)	7.1 (2.1–12.2)
15–24y	100.8	128.5	201.9	135.6	8.0 (6.3–9.6)	5.7 (0.8–10.9)	12.3 (7.9–16.9)
25–34y	84.3	89.0	133.6	82.8	4.3 (2.6–6.0)	1.8 (−3.1 to 7.0)	9.8 (5.2–14.7)
35–64y	63.8	84.6	107.9	74.3	6.2 (5.0–7.3)	5.5 (2.0–9.2)	6.7 (3.7–9.8)
65 + y	40.1	48.2	77.8	40.0	4.7 (2.5–7.1)	7.3 (0.1–14.9)	11.5 (5.2–18.2)

95% CI, 95% confidence interval.

a Trends are shown for 2010–2019 only, as data for 2020 was incomplete and was likely impacted by COVID-19 lockdowns.

### Trends in total anaphylaxis

From 2010 to 2019, anaphylaxis event rates for all causes increased (5.3% per year, 95% CI 4.8–5.8) (Table 2). This increase was greater in females (6.2% per year, 95% CI 5.5–6.9) compared to males (4.3% per year 95% CI 3.6–5.0). Children aged 5–14 years and young and adults aged 15–24 years showed the highest increase (6.9% per year 95% CI 5.6–8.1) and (6.8% per year 95% CI 5.6–8.0) respectively, mostly driven by trends in females. The average annual percent increase in rates was twice as high in the period 2015 to 2019 compared to 2010 to 2014 (6.6% compared to 2.7% per year).

### Distribution of cause-specific anaphylaxis event rates

**Fig. 3:**
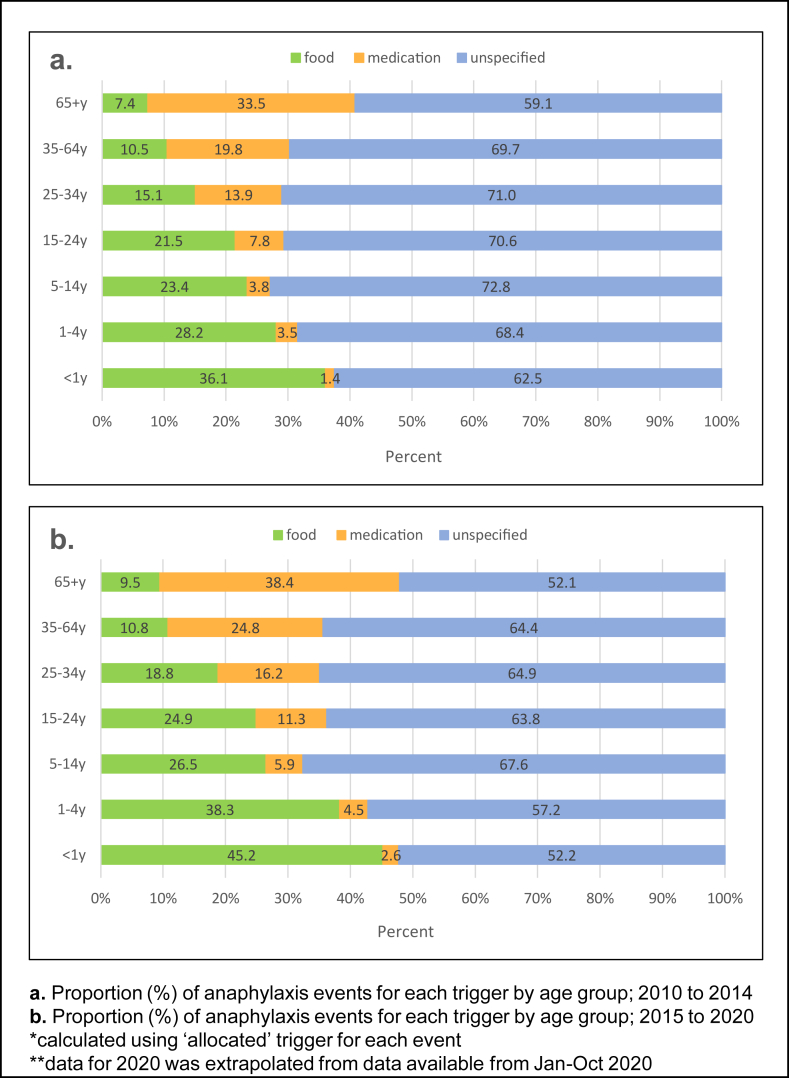
Proportion (%) of anaphylaxis events for each trigger by age group.

Unspecified anaphylaxis was recorded in between 59.1% and 72.8% of events from 2010 to 2014 and between 52.1 and 67.6% in 2015–2020 (Fig. 3). The proportion of food-induced anaphylaxis was highest in infants and decreased with increasing age and medication related anaphylaxis increased with age across the 2 time periods.

### Average annual cause-specific anaphylaxis event rates

From 2010 to 2019, cause-specific event rates all increased (Food: 13.7 to 21.4 per 100 000; Medication: 12.4 to 14.4; unspecified: 44.3 to 78.1), with a drop in rates observed in 2020. Food-related anaphylaxis was highest in infants under 1 year and 1–4 years and lowest in those over 64 years (104.9, 52.8 and 7.1 per 100 000, respectively; 2019). Conversely, medication-related anaphylaxis was highest in those over 65 years and lowest in 5–14 years (22.6 and 2.9 per 100 000, respectively; 2019). (See Supplementary Material Table S4, Figure S1 for cause-specific event rates).

### Trends in cause-specific anaphylaxis

Trends in food-related anaphylaxis show non-significant increases from 2010 to 2014 compared to an increase of 5.0% (95% CI 0.7–9.5) and 10.2% (95% CI 5.9–14.8) annual increase in males and females respectively from 2015 to 2019, driven by teenagers aged above 15 years and adults. (Table 3). There was a non-significant change in rates in infants under 1 year, whilst rates in children 1–4 years have decreased (−3.8% per year 95% CI -6.9 to −0.6) in males and remained stable in females. Rates in children aged 5–14 years have increased in both males (3.8% per year 95% CI 0.8–7.0) and females (7.4% per year 95% CI 3.5–11.5) over the whole study period. A significant change in medication-related rates was observed in females only. Trends in anaphylaxis recorded as unspecified have increased across age groups in line with total anaphylaxis. An acceleration of rates is seen in male (6.9% per year 95% CI 0.3–14.0) and female (12.7% per year 95% CI 3.0–23.3) children aged 1–4 years from 2015 to 2019, compared to non-significant increases from 2010 to 2014.

**Table 3:** Age-standardised trends for cause-specific anaphylaxis event rates

	Average annual % change (95% CI)^a^					
	2010–2019		2010–2014		2015–2019	
	Males	Females	Males	Females	Males	Females
**Food**						
all ages	1.8 (0.3–3.4)	4.7 (3.2–6.3)	−1.5 (−5.9 to 3.0)	2.7 (−1.9 to 7.5)	5.0 (0.7–9.5)	10.2 (5.9–14.8)
<1y	−2.0 (−7.3 to 3.7)	4.4 (−2.5 to 11.7)	−13.9 (−26.8 to 1.1)	−0.9 (−19.1 to 21.3)	4.1 (−11.2 to 22.2)	11.5 (−7.1 to 33.8)
1-4y	−3.8 (−6.9 to −0.6)	1.4 (−3.2 to 6.2)	−3.4 (−11.8 to 5.8)	−3.2 (−15.4 to 10.8)	−3.3 (−12.2 to 6.6)	8.1 (−4.9 to 22.9)
5-14y	3.8 (0.8–7.0)	7.4 (3.5–11.5)	7.3 (−2.2 to 17.7)	1.9 (−9.7 to 14.8)	−0.5 (−8.1 to 7.8)	3.5 (−5.7 to 13.5)
15-24y	3.9 (0.2–7.8)	4.7 (1.5–8.1)	−2.2 (−12.3 to 9.0)	0.4 (−8.6 to 10.3)	10.7 (0.2–22.4)	12.0 (2.8–22.1)
25-34y	4.0 (−0.8 to 9.1)	0.0 (−3.8 to 3.9)	−8.2 (−20.5 to 5.9)	2.4 (−8.1 to 14.2)	16.7 (2.6–32.7)	9.5 (−1.7 to 22.1)
35-64y	5.4 (1.2–9.8)	7.0 (3.7–10.3)	−0.8 (−12.5 to 12.4)	8.7 (−1.3 to 19.7)	11.0 (−0.4 to 23.7)	11.0 (2.4–20.4)
65 + y	0.0 (−8.1 to 8.8)	7.7 (0.2–15.9)	4.8 (−18.1 to 34.1)	9.4 (−12.6 to 37.0)	12.9 (−11.3 to 43.8)	35.6 (10.9–65.8)
**Medication**						
all ages	−0.7 (−2.5 to 1.2)	2.9 (1.4–4.5)	−4.0 (−9.2 to 1.5)	7.9 (3.2–12.9)	−4.7 (−9.6 to 0.5)	4.9 (0.7–9.3)
0-4y^a^	−2.3 (−10.9 to 7.2)	−2.3 (−12.6 to 9.3)	0.5 (−22.0 to 29.5)	−5.6 (−32.5 to 32.2)	14.6 (−13.5 to 51.8)	−25.8 (−46.1 to 2.2)
5-14y	−4.4 (−11.1 to 2.9)	3.4 (−4.5 to 11.8)	−13.0 (−29.9 to 7.9)	32.9 (4.7–68.6)	−18.3 (−33.8 to 0.9)	10.3 (−12.0 to 38.4)
15-24y	−5.0 (−10.7 to 1.0)	3.5 (−1.2 to 8.4)	1.5 (−14.1 to 20.0)	8.4 (−5.4 to 24.2)	−24.5 (−37.6 to −8.6)	4.0 (−8.5 to 18.3)
25-34y	1.3 (−4.1 to 7.1)	3.2 (−0.7 to 7.3)	−5.5 (−19.7 to 11.1)	1.1 (−9.8 to 13.3)	4.7 (−9.8 to 21.6)	12.7 (1.6–25.0)
35-64y	0.4 (−2.4 to 3.3)	2.3 (0.2–4.5)	−4.6 (−12.3 to 3.8)	9.5 (2.8–16.6)	−1.1 (−8.6 to 7.0)	1.2 (−4.6 to 7.2)
65 + y	−0.5 (−4.4 to 3.6)	4.4 (0.8–8.2)	−2.7 (−13.9 to 10.0)	5.6 (−5.4 to 18.0)	−7.0 (−16.5 to 3.6)	11.6 (1.6–22.6)
**Unspecified**						
all ages	5.9 (8.8–6.8)	7.7 (16.16–8.6)	2.6 (0.1–5.3)	3.9 (1.2–6.7)	5.6 (3.4–7.8)	9.4 (7.1–11.8)
<1y	6.2 (1.1–11.6)	7.9 (9.9–14.0)	0.2 (−14.3 to 17.1)	1.0 (−15.3 to 20.4)	1.0 (−10.9 to 14.5)	4.0 (−9.4 to 19.4)
1-4y	6.2 (2.2–8.8)	4.5 (10.10–8.1)	0.4 (−7.2 to 8.6)	0.4 (−9.2 to 11.0)	6.9 (0.3–14.0)	12.7 (3.0–23.3)
5-14y	6.6 (3.3–8.6)	10.2 (11.11–12.8)	6.5 (0.6–12.8)	4.7 (−3.0 to 13.0)	4.4 (−0.3 to 9.4)	8.2 (2.3–14.4)
15-24y	6.9 (4.4–9.2)	10.0 (12.12–12.1)	1.3 (−5.1 to 8.2)	7.4 (1.1–14.2)	7.6 (1.8–13.7)	13.6 (8.2–19.3)
25-34y	6.8 (5.5–9.1)	5.8 (13.13–8.0)	2.9 (−3.9 to 10.2)	1.8 (−4.5 to 8.5)	7.7 (1.8–13.9)	9.2 (3.6–15.1)
35-64y	4.1 (6.6–5.6)	7.7 (14.14–9.2)	2.5 (−1.9 to 7.0)	2.8 (−1.7 to 7.6)	3.1 (−0.7 to 7.1)	8.0 (4.2–12.0)
65 + y	8.2 (7.7–11.7)	4.4 (15.15–7.7)	−1.0 (−10.8 to 9.9)	8.1 (−1.8 to 18.9)	11.3 (3.0–20.3)	7.8 (−0.5 to 16.8)

95% CI, 95% confidence interval.

a Trends are shown for 2010–2019 only, as data for 2020 was incomplete and was likely impacted by COVID-19 lockdowns.

### Health service differences

**Fig. 4:**
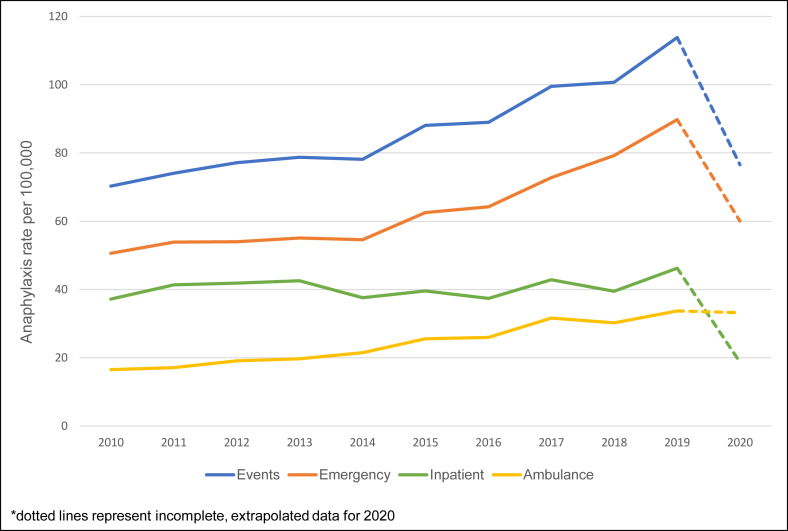
Rate of anaphylaxis by health setting; 2010–2020.

From 2010 to 2019, rates of ambulance and emergency presentations for anaphylaxis have grown by 8.9% (95%CI 7.9–9.8%) and 6.6% per year (95%CI 6.0–7.2%), respectively, hospitalisations appear to be steadying with only a 0.9% (95%CI 0.2–1.6%) annual rise (Fig. 4).

## Discussion

This contemporary study of more than 35 000 health interactions for anaphylaxis provides an overview of the current burden and healthcare utilisation for anaphylaxis in the population. Event rates for anaphylaxis have increased in WA from 2010 to 2019 across all ages, reducing in 2020. These results extend previous published rates in WA, which reported anaphylaxis rates of 82.5 per 100 000 population in 2013.^5^ The current study has shown a further increase to 113.9 per 100 000 in 2019. These rates continue to be higher than national and global estimates for anaphylaxis. Population-based studies from Australia, Asia, the United States, Europe, and the United Kingdom across similar time frames, indicate rates for total anaphylaxis of between 4 and 76 per 100 000 person-years.^10^^,^^11^^,^^14^, ^15^, ^16^, ^17^ These are mostly based on inpatient and emergency data. Inherent issues with the collection and interpretation of anaphylaxis data means that making any direct comparisons between populations is open to misinterpretation. Variation in clinical definitions, coding issues in health datasets, ability to capture cases and different demographic profile of populations as well as epidemiological measures, all have an impact on burden estimates.^2^ The inclusion of ambulance, emergency, and inpatient linked data in this study allowed more comprehensive capture of cases; therefore, higher rates are to be expected and estimates are likely to be more robust. Even so, when comparing published rates from around the world using single health datasets, we still found higher rates in WA than elsewhere.^11^^,^^14^^,^^17^^,^^18^ It is hard to determine if disease burden is actually higher in the WA population or whether there are important differences in diagnosis, management, or health seeking behaviour. Even within the state, there are differences in health care access across regions. The observation that a higher proportion of anaphylaxis events occurred in less socially disadvantaged regions (which tend to be in major cities) may reflect variation in rural and remote versus urban management of anaphylaxis.

Trends in anaphylaxis event rates varied by age and gender in WA. Gender differences were dependent on age with rates highest in male children under 15 years and higher in females older than 15 years. This is in line with previous published studies.^5^^,^^19^^,^^20^ However, much of the increase in anaphylaxis has been driven by rates in females across age groups. Over the whole study period, anaphylaxis events have increased by 5.3% per year and the rate of change was greatest in those aged 5–24 years. When examining early and contemporary changes, it appears that trends in anaphylaxis are accelerating. The rate of annual increase was 2.7% per year from 2010 to 2014 and 6.6% from 2015 to 2019, which was dominated by trends in ages 15–24 and 25–34 years. This acceleration in annual rate of increase was greater in females, with a 12.3% rise per year in 15–24 year olds. Increasing trends in adolescents and young adults observed may be due to persisting risk of anaphylaxis in the aging cohort or an increase in adult-onset anaphylaxis. Regardless, it is important to monitor this population as adolescents and young adults are at greatest risk of severe outcomes including death.^21^ Challenges faced in transitioning from the care of parents and childhood allergists and learning how to self-manage risk are important to inform prevention strategies and optimal emergency treatment in this age group.^22^

Reports from international epidemiological and registry studies have also highlighted the growing problem of anaphylaxis in older adults.^8^^,^^23^^,^^24^ Our study shows high rates (range: 71.9–114.8 per 100 000; 2019) in adults aged over 25 years. The clinical characteristics and causes of anaphylaxis vary between adults and children, with past research highlighting adults having more cardiovascular symptoms and reactions to insect sings and medication.^8^^,^^23^^,^^25^^,^^26^ Whilst medication-related anaphylaxis rates in our study are higher in older adults, compared to food, trends have remained relatively stable, only increasing significantly in females over 35 years. In comparison, trends in food-related anaphylaxis has increased in both adolescents and adults. Older adults are more likely to have atypical clinical presentation, increased comorbidities and receive delayed adrenaline, increasing their risk for severe and fatal reactions.^8^^,^^23^ Indeed, almost all anaphylaxis deaths occurred in adults over the age of 30 years.

The greatest burden of anaphylaxis remains in infants. Food is most commonly implicated in infant and childhood anaphylaxis. Of note, rates of infant food anaphylaxis in our study (149.1 per 100 000 in 2015) are higher than New Zealand^27^ and United States^28^ with rates of 50.5 in 2015 and 27.3 per 100 000 in 2014, respectively in under two-year olds. WA rates in 2019 (104.9 per 100 000) are also slightly higher than Australian estimates (74.3 per 100 000; 2019–2020), based on national morbidity data.^6^ Variability in rates for infant food anaphylaxis may be in part due to differences in diagnosis criteria between countries.^19^ Monitoring trends in this vulnerable group, is vital as anaphylaxis is frequently under-diagnosed and therefore mismanaged.^29^

In light of recent changes to global infant feeding guidelines (including Australia), there is much interest on the potential reduction in food-related anaphylaxis in later years.^30^ Mullins et al have recently published findings that may point to a slowing of year-on-year rate of increase in food anaphylaxis among those aged 1–4, 5–9 and 10–14 years, coinciding with the introduction of updated infant feeding and allergy prevention guidelines in 2008 and 2016.^6^ Our data highlight a non-significant decline in food anaphylaxis for ages 1–4 years overall, with a significant decline in male infants (−3.8% per year) from 2010 to 2019. Trends in ages 5–14 years have increased (3.8% in males and 7.4% per year in females) overall, with no significant change seen across the 2 five-year time-periods. In comparison, trends in adults, 15 years and over show non-significant changes in rates early on followed by large increases (between 11 and 35.6% per year) from 2015 to 2019. Whilst this does point to a potential steadying of food-related anaphylaxis rates in children compared to increases in adults, it is important to highlight the background increase in unspecified anaphylaxis across age groups.

Trends in total anaphylaxis were underpinned by increases in unspecified anaphylaxis. There is more clarity around cause in the very old and the very young, showing a smaller proportion of recorded as unspecified. However, between 52% and 73% of anaphylaxis events are recorded as unspecified across age groups. The high proportion of events recorded as unspecified means that we have to interpret cause-specific anaphylaxis trends with caution. Whilst food and medication-related anaphylaxis trends provide insight into changes in the demographic profile of people at risk of anaphylaxis due to these triggers, which has implications for allergy prevention and management of reactions, the increase in total anaphylaxis rates highlights the continued growing burden that anaphylaxis poses to the community. Events recorded as unspecified may indicate that patients are either unaware of their trigger, stressing the need for better allergy testing as many may face long wait lists for allergy/immunology specialist diagnosis, or have come into contact with an unknown or undeclared allergen, which has further implications for public health safety.

Anaphylaxis being treated by ambulance and emergency have increased (8.9% and 6.6% per year, respectively) whereas hospital admissions have remained relatively steady. Whilst emergency presentation and admission rates dropped from 2019 to 2020, ambulance rates stayed high. In comparison, ambulance, emergency and inpatient presentation/admissions for any health reason, declined during this time. The decrease in rates in 2020 is likely due to the impact that the COVID-19 pandemic and associated lockdowns in WA had on the health-seeking behaviour of people experiencing anaphylaxis, with many treated in ambulance either following health advice or refusing to be taken to hospital. Even when considering total anaphylaxis, rates dropped from 113.9 per 100 000 in 2019 to 76.5 per 100 000 in 2020. Beside a reluctance to seek medical treatment in the hospital, the inability to eat out at restaurants, visit friends and attend school during periods of lockdown could be one explanation for this reduction in anaphylaxis overall.

### Strengths and limitations

As with all epidemiological studies that utilise routinely collected health data to report anaphylaxis, the lack of appropriate coding for anaphylaxis is an important limitation. A recent validation study estimated that the positive predictive value of ICD-10 codes was only 64% and the sensitivity of emergency department and inpatient anaphylaxis coding for all validated events was 58%.^31^ As no validation of any coded cases was not done in this study it is not possible to determine the importance of miscoded anaphylaxis. The lack of clinical information in our dataset means we cannot describe the symptoms or severity of anaphylaxis. However, clinical review of records is time intensive so there will always be a trade-off between accuracy and capturing a sufficient number of events. The high number of anaphylaxis interactions in our study allow investigation of population trends, not achievable in smaller studies. Whilst this study likely still underestimates the true burden of anaphylaxis in the WA population, it is strengthened by the use of linked health data, which provides a more comprehensive method of capturing cases including those occurring in the prehospital setting. Still, the inability to capture cases that occur in the community means that our rates will only capture more severe events.

The high proportion of events recorded as unspecified poses an issue with interpretability of cause-specific distribution of events. This is true for all studies using ICD codes; however, it still allows cause-specific rates to be compared with past research. Additionally, there is no cause-specific anaphylaxis code in the ambulance dataset which means all events (that are not contingent with a cause-specific anaphylaxis emergency or hospital record) were counted as unspecified. Inherent issues with coding deaths due to anaphylaxis is well established.^2^ Anaphylaxis is poorly reported in mortality records.^32^ Our study recorded 13 anaphylaxis deaths, none of which had an ICD code for anaphylaxis as the primary cause and most were recorded as unspecified in the secondary cause of death field. This highlights the need for better reporting of anaphylaxis reactions, including deaths, through a structured reporting system.

## Conclusion

This study has provided contemporary information on the burden and healthcare utilisation of anaphylaxis patients in WA from 2010 to 2020. Rates of anaphylaxis continue to increase, with WA having higher rates than previous estimates for Australia. Whilst rates are still high in infants, the lower rates in children compared to older age groups is important to monitor. This may indicate better prevention of allergy or increases in adult-onset anaphylaxis, which have important implications for education and management in this age group. Emergency presentations and prehospital treatment of anaphylaxis by ambulance has continued to rise. Exploration of the patient care journey, from prehospital to in-hospital care, will inform better management and appropriate access to care.

## Abbreviation

WA. Western Australia; ACE-WA, Anaphylaxis Epidemiology and Characteristics in Western Australia; WADLS, Western Australian Data Linkage System; SJA, St John Ambulance; ICD, International Classification of Diseases; ICD-10-AM, International Statistical Classification of Diseases and Related Health Problems 10th revision Australian Modification; SEIFA, Socio-Economic Indexes for Areas; IRSAD, Index of Relative Socio-Economic Advantage and Disadvantage; US, United States; UK, United Kingdom; ED, Emergency Department

## Acknowledgments

The authors thank staff at the Western Australian Data Linkage Branch and data custodians of the Western Australian Department of Health Hospital Morbidity Data, Emergency Department Data Collections and Registrar General for access to and provision of the State linked data. We thank St John Ambulance Western Australia for providing data for linkage and Dr Paul Bailey who reviewed the study and provided approval for ambulance data. We also thank Dr Ross Marriott and Associate Professor Kevin Murray from the University of Western Australia, for their early input to the design of the study and statistical review.

## Funding

The research was supported by a research grant provided by the Western Australian Department of Health (G05767) and an unrestricted grant provided by Mylan Specialty Limited (IIT16-007).

## Availability of data and materials

The Western Australian Department of Health approvals for data forbid release of linked data publicly, in line with privacy laws. Therefore, data for this project is not available for release.

## Authors’ contributions

SLS co-designed, completed data collection and analysis, interpreted the results, wrote the first draft and prepared the final version of the manuscript. FS provided expertise on study design and supervision, assisted with data analysis, interpreted results, and reviewed manuscript. RL provided clinical perspective, interpreted the results and reviewed the manuscript. MS provided consumer and clinical perspective, interpreted the results and reviewed the manuscript. RC interpreted the results and reviewed the manuscript. SMS raised funding for the study, supervised and co-designed the study, assisted with data analysis, interpreted results, and reviewed manuscript.

## Ethics statement

Approval to conduct this research was obtained from the Human Research Ethics Committees of the Western Australian Department of Health and The University of Western Australia.

## Consent for publication

All authors consent to this work being published.

## Competing interest

The authors report no competing interests.

## Submission declaration

The work described has not been published previously, is not under consideration for publication elsewhere, its publication is approved by all authors.
