# Supplementary material for: Contemporary trends in anaphylaxis burden and healthcare utilisation in Western Australia: A linked data study
Source: World Allergy Organ J. 2023 Sep 27;16(9):100818. doi: 10.1016/j.waojou.2023.100818 (PMC10534263; doi:10.1016/j.waojou.2023.100818)

**Supplementary material**

Table S1. Diagnosis codes used to identify the anaphylaxis analysis cohort.

Table S2. Age-standardised trends for cause-specific anaphylaxis event rates from 2010 to 2020.

Table S3. Primary cause of death listed for anaphylaxis deaths.

Table S4. Age-standardised anaphylaxis event rates, 2010 to 2020.

Figure S1. Age-standardised anaphylaxis event rates by cause per 100,000 person-years, 2010 to 2020.

Table S1. Diagnosis codes used to identify the anaphylaxis analysis cohort.

The codes relevant to this analysis cohort (2010 to 2020) are ICD-10-AM (International Statistical Classification of Diseases and Related Health Problems 10th revision Australian Modification) codes, St John Ambulance Problem code and Emergency Department Dataset Collection Symptom code specific to anaphylaxis.

Anaphylaxis presentations were identified in the principal diagnosis fields in the emergency dataset, principal and secondary diagnoses fields in the inpatient hospital dataset, and problem field in the ambulance dataset. Anaphylaxis deaths were identified from the underlying ICD coded cause of death as well as free text field using “anaph” as the search string in the Death Register dataset.

|  |  | **Dataset** | | | |
| --- | --- | --- | --- | --- | --- |
| **code** | **code description** | **Ambulance** | **Emergency** | **Inpatient** | **Death** |
| ***ICD10-AM code*** | | | | | |
| T78.0 | anaphylactic shock due to adverse food reaction | NA | 407 | 4126 | 2 |
| T88.6 | anaphylactic shock due to adverse event of correct drug or medicament properly administered | NA | 1382 | 2678 | 2 |
| T78.2 | anaphylactic shock not otherwise specified | NA | 15810 | 3859 | 9 |
| ***Ambulance problem code*** | | | | | |
| 221 | anaphylaxis | 6879 | NA | NA | NA |
| ***Emergency symptom code*** | | | | | |
| BFB00 | anaphylaxis | NA | 4 | NA | NA |
|  |  | **6879** | **17603** | **10663** | **13** |

Table S2. Age-standardised trends for cause-specific anaphylaxis event rates from 2010 to 2020.

|  | **Average annual % change (95% CI)*** | | | | | | | |
| --- | --- | --- | --- | --- | --- | --- | --- | --- |
|  | **2010 - 2020** | |  | **2010 - 2014** | |  | **2015 - 2020** | |
|  | **Males** | **Females** |  | **Males** | **Females** |  | **Males** | **Females** |
| **All-cause** |  |  |  |  |  |  |  |  |
| all ages | 2.5 (1.9 to 3.1) | 4.1 (3.6 to 4.7) |  | 0.9 (-1.2 to 3.0) | 4.5 (2.4 to 6.7) |  | -1.1 (-2.5 to 0.2) | 1.8 (0.5 to 3.2) |
| <1y | 1.8 (-1.3 to 5.1) | 4.2 (0.5 to 8.1) |  | -6.5 (-16.2 to 4.4) | -1.2 (-13.4 to 12.6) |  | -0.1 (-7.3 to 7.6) | 0.6 (-7.5 to 9.4) |
| 1-4y | 1.9 (0.2 to 3.6) | 1.7 (-0.6 to 4.1) |  | -1.2 (-6.8 to 4.7) | -0.7 (-8.2 to 7.5) |  | 2.1 (-1.9 to 6.2) | 2.0 (-3.4 to 7.7) |
| 5-14y | 3.1 (1.8 to 4.5) | 6.3 (4.6 to 8.0) |  | 5.7 (0.8 to 10.8) | 5.7 (-0.6 to 12.5) |  | -2.9 (-5.8 to 0.1) | 0.3 (-3.2 to 4.0) |
| 15-24y | 3.6 (2.0 to 5.1) | 6.2 (4.8 to 7.6) |  | 0.5 (-4.7 to 6.0) | 5.7 (0.8 to 10.9) |  | 0.6 (-2.9 to 4.3) | 5.4 (2.3 to 8.7) |
| 25-34y | 3.5 (1.8 to 5.1) | 2.5 (1.0 to 3.9) |  | -0.1 (-5.7 to 5.9) | 1.8 (-3.1 to 7.0) |  | 0.9 (-2.8 to 4.8) | 2.4 (-0.9 to 5.9) |
| 35-64y | 1.6 (0.6 to 2.7) | 4.1 (3.1 to 5.1) |  | 0.8 (-2.8 to 4.6) | 5.5 (2.0 to 9.2) |  | -2.4 (-4.9 to 0.0) | 0.6 (-1.6 to 2.8) |
| 65+y | 1.6 (-0.4 to 3.7) | 1.9 (0.0 to 3.9) |  | -1.1 (-8.3 to 6.7) | 7.3 (0.1 to 14.9) |  | -3.1 (-7.5 to 1.6) | 0.9 (-3.4 to 5.5) |
| **Food** |  |  |  |  |  |  |  |  |
| all ages | -1.1 (-2.5 to 0.2) | 1.9 (0.6 to 3.3) |  | -1.5 (-5.9 to 3.0) | 2.7 (-1.9 to 7.5) |  | -5.3 (-8.4 to -2.1) | -0.2 (-3.3 to 3.0) |
| <1y | -2.7 (-7.4 to 2.2) | 1.8 (-4.1 to 8.0) |  | -13.9 (-26.8 to 1.1) | -0.9 (-19.1 to 21.3) |  | -1.1 (-12.6 to 11.9) | 0.9 (-12.4 to 16.2) |
| 1-4y | -5.4 (-8.1 to -2.6) | -1.7 (-5.6 to 2.4) |  | -3.4 (-11.8 to 5.8) | -3.2 (-15.4 to 10.8) |  | -8.6 (-15.3 to -1.3) | -4.1 (-13.4 to 6.2) |
| 5-14y | -0.2 (-2.8 to 2.5) | 3.6 (0.3 to 6.9) |  | 7.3 (-2.2 to 17.7) | 1.9 (-9.7 to 14.8) |  | -10.5 (-16.0 to -4.6) | -5.7 (-12.4 to 1.4) |
| 15-24y | 2.2 (-0.9 to 5.5) | 2.6 (-0.1 to 5.5) |  | -2.2 (-12.3 to 9.0) | 0.4 (-8.6 to 10.3) |  | 2.7 (-4.9 to 10.8) | 2.7 (-3.9 to 9.7) |
| 25-34y | 1.0 (-3.2 to 5.3) | -1.1 (-4.4 to 2.2) |  | -8.2 (-20.5 to 5.9) | 2.4 (-8.1 to 14.2) |  | 2.1 (-7.6 to 12.9) | 2.4 (-5.8 to 11.2) |
| 35-64y | -0.3 (-3.8 to 3.4) | 3.8 (1.0 to 6.6) |  | -0.8 (-12.5 to 12.4) | 8.7 (-1.3 to 19.7) |  | -8.1 (-15.8 to 0.4) | 0.4 (-5.7 to 6.9) |
| 65+y | -4.9 (-11.8 to 2.6) | 1.2 (-5.0 to 7.9) |  | 4.8 (-18.1 to 34.1) | 9.4 (-12.6 to 37.0) |  | -7.5 (-23.8 to 12.3) | 4.2 (-10.6 to 21.4) |
| **Medication** | |  |  |  |  |  |  |  |
| all ages | -3.2 (-4.8 to -1.5) | -0.6 (-1.9 to 0.7) |  | -4.0 (-9.2 to 1.5) | 7.9 (3.2 to 12.9) |  | -11.0 (-14.7 to -7.2) | -6.2 (-9.2 to -3.1) |
| 0-4y* | -5.9 (-13.5 to 2.4) | -4.1 (-13.2 to 5.9) |  | 0.5 (-22.0 to 29.5) | -5.6 (-32.5 to 32.2) |  | -4.2 (-23.6 to 20.0) | -23.9 (-41.1 to -1.7) |
| 5-14y | -5.8 (-11.7 to 0.5) | 0.5 (-6.2 to 7.7) |  | -13.0 (-29.9 to 7.9) | 32.9 (4.7 to 68.6) |  | -18.5 (-31.0 to -3.7) | -0.2 (-16.2 to 18.9) |
| 15-24y | -7.7 (-12.7 to -2.4) | 0.3 (-3.7 to 4.5) |  | 1.5 (-14.1 to 20.0) | 8.4 (-5.4 to 24.2) |  | -27.1 (-37.9 to -14.4) | -5.6 (-14.7 to 4.5) |
| 25-34y | -0.2 (-4.9 to 4.7) | -0.1 (-3.4 to 3.3) |  | -5.5 (-19.7 to 11.1) | 1.1 (-9.8 to 13.3) |  | -1.5 (-12.2 to 10.6) | -0.9 (-8.7 to 7.5) |
| 35-64y | -1.6 (-4.0 to 0.9) | -1.3 (-3.1 to 0.6) |  | -4.6 (-12.3 to 3.8) | 9.5 (2.8 to 16.6) |  | -6.7 (-12.3 to -0.8) | -9.2 (-13.3 to -4.8) |
| 65+y | -4.7 (-8.0 to -1.2) | 0.6 (-2.4 to 3.8) |  | -2.7 (-13.9 to 10.0) | 5.6 (-5.4 to 18.0) |  | -17.3 (-24.3 to -9.6) | -2.2 (-9.1 to 5.2) |
| **Unspecified** | |  |  |  |  |  |  |  |
| all ages | 4.4 (3.7 to 5.1) | 6.2 (5.4 to 7.0) |  | 2.6 (0.1 to 5.3) | 3.9 (1.2 to 6.7) |  | 1.3 (-0.2 to 3.0) | 4.4 (2.8 to 6.1) |
| <1y | 5.8 (1.4 to 10.3) | 6.1 (1.2 to 11.3) |  | 0.2 (-14.3 to 17.1) | 1.0 (-15.3 to 20.4) |  | 1.4 (-7.7 to 11.5) | 0.4 (-9.6 to 11.6) |
| 1-4y | 6.3 (4.0 to 8.5) | 3.8 (0.9 to 6.8) |  | 0.4 (-7.2 to 8.6) | 0.4 (-9.2 to 11.0) |  | 6.6 (1.7 to 11.9) | 7.2 (0.2 to 14.7) |
| 5-14y | 4.9 (3.3 to 6.5) | 7.8 (5.7 to 10.0) | | 6.5 (0.6 to 12.8) | 4.7 (-3.0 to 13.0) |  | 0.4 (3.0 to 4.0) | 0.4 (-3.0 to 4.0) |
| 15-24y | 5.3 (3.4 to 7.2) | 8.4 (6.7 to 10.2) | | 1.3 (-5.1 to 8.2) | 7.4 (1.1 to 14.2) |  | 2.5 (-1.7 to 6.9) | 2.5 (-1.7 to 6.9) |
| 25-34y | 4.5 (2.6 to 6.5) | 4.2 (2.4 to 6.0) |  | 2.9 (-3.9 to 10.2) | 1.8 (-4.5 to 8.5) |  | 1.1 (-3.2 to 5.5) | 3.2 (-0.9 to 7.4) |
| 35-64y | 2.6 (1.4 to 3.9) | 6.4 (5.1 to 7.7) |  | 2.5 (-1.9 to 7.0) | 2.8 (-1.7 to 7.6) |  | -0.9 (-3.7 to 2.0) | 4.2 (1.4 to 7.0) |
| 65+y | 5.9 (3.1 to 8.8) | 3.0 (0.3 to 5.7) |  | -1.0 (-10.8 to 9.9) | 8.1 (-1.8 to 18.9) |  | 3.7 (-2.2 to 9.9) | 2.6 (-3.4 to 9.0) |

95% CI, 95% confidence interval.

Table S3. Primary cause of death listed for anaphylaxis deaths.

| **Primary Cause of Death Code** | **Code description** | **Frequency** |
| --- | --- | --- |
| X23 | Contact with hornets, wasps and bees | 2 |
| X23.0 | Contact with hornets, wasps and bees, home | 1 |
| X25.4 | Contact with other specified venomous arthropods, street and highway | 1 |
| X58 | Exposure to other specified factors | 1 |
| Y57.9 | Complications due to medicines or drugs | 3 |
| J45.9 | Asthma, unspecified | 1 |
| J18.9 | Pneumonia, unspecified | 1 |
| B18.2 | Chronic viral hepatitis C | 1 |
| C26.0 | Malignant neoplasm of intestinal tract, part unspecified | 1 |
| L03.8 | Cellulitis of other sites | 1 |
|  |  | 13 |

Table S4. Age-standardised anaphylaxis event rates, 2010 to 2020

| *Age-standardised annual event rates per 100 000 population* | | | | | | | | | | | | |
| --- | --- | --- | --- | --- | --- | --- | --- | --- | --- | --- | --- | --- |
| **Age (years)** | **2010** | | **2011** | **2012** | **2013** | **2014** | **2015** | **2016** | **2017** | **2018** | **2019** | **2020** |
| **All-cause** |  | |  |  |  |  |  |  |  |  |  |  |
| **All persons** |  | |  |  |  |  |  |  |  |  |  |  |
| **all ages** | 70.3 | | 74.1 | 77.2 | 78.8 | 78.2 | 88.1 | 89.0 | 99.6 | 100.7 | 113.9 | 76.5 |
| **<1y** | 153.6 | | 220.8 | 136.6 | 171.3 | 141.7 | 195.0 | 223.9 | 212.7 | 159.5 | 269.7 | 181.4 |
| **1-4y** | 138.2 | | 133.4 | 147.4 | 132.0 | 132.3 | 137.0 | 151.5 | 153.5 | 153.8 | 179.9 | 142.5 |
| **5-14y** | 71.7 | | 98.4 | 90.6 | 86.2 | 102.6 | 121.4 | 115.9 | 135.0 | 132.0 | 140.1 | 95.0 |
| **15-24y** | 84.8 | | 88.6 | 92.4 | 97.1 | 95.9 | 113.6 | 110.2 | 118.2 | 135.0 | 158.4 | 109.7 |
| **25-34y** | 70.8 | | 72.8 | 78.0 | 74.6 | 74.2 | 77.7 | 83.7 | 94.8 | 93.6 | 114.8 | 70.8 |
| **35-64y** | 63.0 | | 58.2 | 66.0 | 71.6 | 66.8 | 76.3 | 77.1 | 84.9 | 87.9 | 91.9 | 63.5 |
| **65+y** | 44.2 | | 49.6 | 50.9 | 52.8 | 51.3 | 52.4 | 50.5 | 67.4 | 61.3 | 71.9 | 39.2 |
| **Males** |  | |  |  |  |  |  |  |  |  |  |  |
| **all ages** | 71.3 | | 76.0 | 75.4 | 76.6 | 74.4 | 88.6 | 85.6 | 98.3 | 95.5 | 103.7 | 71.8 |
| **<1y** | 186.9 | | 245.8 | 165.6 | 209.0 | 141.9 | 234.5 | 238.4 | 205.4 | 178.3 | 284.5 | 211.6 |
| **1-4y** | 176.2 | | 156.1 | 199.4 | 181.7 | 153.9 | 182.5 | 189.7 | 188.8 | 198.8 | 218.4 | 191.5 |
| **5-14y** | 87.9 | | 118.5 | 118.0 | 103.9 | 126.3 | 149.2 | 135.3 | 151.4 | 143.8 | 161.6 | 106.8 |
| **15-24y** | 69.8 | | 90.1 | 75.8 | 86.9 | 73.4 | 99.5 | 90.5 | 103.5 | 113.1 | 117.6 | 85.4 |
| **25-34y** | 58.1 | | 67.4 | 61.6 | 53.3 | 65.1 | 66.9 | 72.9 | 87.2 | 82.6 | 96.0 | 58.9 |
| **35-64y** | 62.2 | | 55.1 | 59.1 | 65.4 | 59.8 | 68.2 | 68.0 | 78.2 | 75.0 | 76.0 | 52.7 |
| **65+y** | 48.8 | | 47.8 | 44.2 | 48.8 | 45.3 | 57.1 | 48.3 | 71.6 | 62.0 | 66.2 | 38.6 |
| **Females** |  | |  |  |  |  |  |  |  |  |  |  |
| **all ages** | 69.5 | | 71.9 | 78.8 | 81.1 | 82.0 | 87.6 | 92.4 | 101.0 | 105.9 | 124.2 | 81.2 |
| **<1y** | 118.4 | | 195.1 | 106.0 | 131.4 | 141.4 | 153.2 | 208.5 | 220.5 | 139.6 | 254.0 | 149.3 |
| **1-4y** | 98.6 | | 109.7 | 93.1 | 79.9 | 109.7 | 89.4 | 111.7 | 116.4 | 106.1 | 138.8 | 90.3 |
| **5-14y** | 54.8 | | 77.5 | 62.2 | 67.7 | 77.7 | 92.2 | 95.6 | 117.8 | 119.5 | 117.5 | 82.5 |
| **15-24y** | 100.8 | | 87.1 | 110.0 | 107.9 | 119.6 | 128.5 | 130.9 | 133.7 | 158.1 | 201.9 | 135.6 |
| **25-34y** | 84.3 | | 78.5 | 95.6 | 97.4 | 83.9 | 89.0 | 94.9 | 102.5 | 104.7 | 133.6 | 82.8 |
| **35-64y** | 63.8 | | 61.4 | 73.1 | 78.1 | 74.0 | 84.6 | 86.4 | 91.9 | 100.9 | 107.9 | 74.3 |
| **65+y** | 40.1 | | 50.8 | 56.0 | 56.4 | 56.6 | 48.2 | 52.5 | 64.6 | 60.3 | 77.8 | 40.0 |
| **Food** |  | |  |  |  |  |  |  |  |  |  |  |
| **All persons** |  | |  |  |  |  |  |  |  |  |  |  |
| **all ages** | 13.7 | | 16.0 | 15.4 | 16.2 | 14.1 | 14.5 | 16.8 | 17.9 | 16.1 | 21.4 | 9.4 |
| **<1y** | 67.2 | | 115.2 | 66.8 | 69.7 | 54.9 | 63.1 | 92.4 | 66.1 | 65.0 | 104.9 | 54.4 |
| **1-4y** | 51.5 | | 60.7 | 50.2 | 52.5 | 47.0 | 43.7 | 48.8 | 53.1 | 34.3 | 52.8 | 23.6 |
| **5-14y** | 19.1 | | 27.9 | 21.6 | 24.0 | 27.0 | 28.5 | 34.6 | 32.4 | 31.9 | 31.8 | 13.2 |
| **15-24y** | 22.6 | | 21.1 | 25.7 | 25.0 | 19.9 | 25.2 | 21.3 | 28.1 | 26.7 | 37.5 | 20.6 |
| **25-34y** | 14.6 | | 13.1 | 14.0 | 15.5 | 12.4 | 10.6 | 13.3 | 16.0 | 11.1 | 20.1 | 9.8 |
| **35-64y** | 5.5 | | 6.6 | 8.3 | 8.0 | 6.5 | 6.9 | 9.0 | 8.9 | 9.4 | 11.5 | 4.1 |
| **65+y** | 3.5 | | 4.5 | 4.3 | 6.6 | 4.3 | 2.7 | 3.4 | 5.3 | 5.4 | 7.1 | .7 |
| **Males** |  | |  |  |  |  |  |  |  |  |  |  |
| **all ages** | 14.2 | | 16.3 | 15.7 | 15.7 | 13.5 | 15.0 | 14.8 | 18.2 | 14.9 | 19.1 | 7.7 |
| **<1y** | 99.7 | | 126.0 | 88.7 | 79.1 | 56.8 | 83.8 | 75.8 | 88.8 | 80.5 | 98.7 | 64.7 |
| **1-4y** | 72.1 | | 74.9 | 74.0 | 68.9 | 63.3 | 62.8 | 57.0 | 67.1 | 38.9 | 62.2 | 31.9 |
| **5-14y** | 23.2 | | 30.2 | 30.1 | 29.2 | 34.2 | 36.1 | 35.6 | 37.8 | 32.8 | 36.7 | 12.7 |
| **15-24y** | 14.8 | | 23.4 | 20.7 | 23.5 | 12.7 | 18.1 | 18.9 | 26.7 | 22.8 | 27.8 | 17.7 |
| **25-34y** | 12.6 | | 9.9 | 9.5 | 8.5 | 9.2 | 9.1 | 9.3 | 13.8 | 8.1 | 18.9 | 6.1 |
| **35-64y** | 4.6 | | 5.1 | 6.4 | 5.3 | 4.2 | 5.3 | 5.6 | 6.8 | 7.3 | 8.0 | 0.7 |
| **65+y** | 4.5 | | 3.6 | 2.7 | 7.0 | 3.7 | 2.5 | 3.0 | 4.9 | 5.3 | 3.6 | 0.4 |
| **Females** |  | |  |  |  |  |  |  |  |  |  |  |
| **all ages** | 13.2 | | 15.7 | 15.1 | 16.8 | 14.7 | 14.1 | 18.7 | 17.5 | 17.4 | 23.7 | 11.1 |
| **<1y** | 32.9 | | 104.1 | 43.6 | 59.7 | 53.0 | 41.3 | 110.0 | 41.7 | 48.6 | 111.5 | 43.6 |
| **1-4y** | 30.1 | | 45.8 | 25.3 | 35.3 | 30.1 | 23.8 | 40.2 | 38.3 | 29.5 | 42.8 | 14.8 |
| **5-14y** | 14.7 | | 25.5 | 12.8 | 18.4 | 19.4 | 20.5 | 33.5 | 26.7 | 30.9 | 26.7 | 13.8 |
| **15-24y** | 30.9 | | 18.7 | 31.1 | 26.7 | 27.5 | 32.8 | 23.8 | 29.5 | 30.9 | 47.9 | 23.7 |
| **25-34y** | 16.7 | | 16.6 | 18.8 | 23.1 | 15.8 | 12.1 | 17.5 | 18.1 | 14.1 | 21.4 | 13.5 |
| **35-64y** | 6.4 | | 8.1 | 10.2 | 10.6 | 8.9 | 8.6 | 12.4 | 11.0 | 11.7 | 15.0 | 7.4 |
| **65+y** | 2.7 | | 5.2 | 5.8 | 6.2 | 4.8 | 2.9 | 3.7 | 5.8 | 5.5 | 10.4 | 0.9 |
| **Medication** |  | |  |  |  |  |  |  |  |  |  |  |
| **All persons** |  | |  |  |  |  |  |  |  |  |  |  |
| **all ages** | 12.4 | | 12.7 | 13.2 | 13.9 | 13.9 | 14.0 | 13.3 | 14.9 | 14.5 | 14.4 | 6.3 |
| **0-4y** | 7.2 | | 4.5 | 5.5 | 6.0 | 5.9 | 5.2 | 5.8 | 6.9 | 4.0 | 4.6 | 1.7 |
| **5-14y** | 5.5 | | 5.3 | 3.7 | 4.5 | 7.3 | 5.0 | 5.0 | 6.0 | 6.2 | 2.9 | 2.8 |
| **15-24y** | 7.7 | | 11.8 | 10.4 | 11.4 | 10.7 | 12.9 | 11.3 | 8.2 | 11.4 | 9.5 | 4.3 |
| **25-34y** | 12.5 | | 10.0 | 14.8 | 11.8 | 11.3 | 10.5 | 13.0 | 12.7 | 13.5 | 16.8 | 7.2 |
| **35-64y** | 16.4 | | 14.4 | 14.0 | 18.4 | 17.7 | 18.4 | 14.5 | 19.7 | 17.5 | 17.3 | 7.9 |
| **65+y** | 14.3 | | 21.8 | 23.0 | 18.0 | 18.5 | 18.8 | 22.3 | 21.5 | 21.6 | 22.6 | 7.7 |
| **Males** |  | |  |  |  |  |  |  |  |  |  |  |
| **all ages** | 11.0 | | 10.5 | 11.2 | 10.1 | 9.4 | 12.5 | 10.2 | 11.4 | 10.3 | 9.9 | 5.0 |
| **0-4y** | 8.9 | | 2.5 | 9.6 | 9.3 | 5.7 | 1.1 | 6.8 | 10.0 | 3.4 | 6.6 | 1.1 |
| **5-14y** | 6.5 | | 6.5 | 5.3 | 5.0 | 3.7 | 7.3 | 5.4 | 6.5 | 5.2 | 2.2 | 2.7 |
| **15-24y** | 6.5 | | 10.0 | 6.9 | 9.1 | 7.5 | 11.1 | 10.0 | 2.3 | 7.8 | 3.0 | 1.8 |
| **25-34y** | 6.8 | | 9.4 | 10.5 | 5.1 | 7.3 | 8.7 | 7.3 | 6.8 | 10.5 | 9.2 | 6.0 |
| **35-64y** | 13.5 | | 11.2 | 10.9 | 11.2 | 11.1 | 13.9 | 9.0 | 14.8 | 12.6 | 11.6 | 7.5 |
| **65+y** | 17.5 | | 17.3 | 21.6 | 17.7 | 15.3 | 23.0 | 21.8 | 19.8 | 13.5 | 19.8 | 4.1 |
| **Females** |  | |  |  |  |  |  |  |  |  |  |  |
| **all ages** | 13.8 | | 14.9 | 15.2 | 17.8 | 18.4 | 15.7 | 16.5 | 18.4 | 18.5 | 19.0 | 7.5 |
| **0-4y** | 5.3 | | 6.5 | 1.3 | 2.4 | 6.0 | 9.5 | 4.7 | 3.5 | 4.7 | 2.4 | 2.4 |
| **5-14y** | 4.4 | | 4.0 | 2.0 | 3.9 | 11.0 | 2.6 | 4.4 | 5.6 | 7.3 | 3.6 | 2.8 |
| **15-24y** | 8.9 | | 13.7 | 14.0 | 13.9 | 14.0 | 14.8 | 12.6 | 14.5 | 15.3 | 16.4 | 6.9 |
| **25-34y** | 18.5 | | 10.6 | 19.4 | 19.1 | 15.5 | 12.5 | 19.0 | 18.7 | 16.5 | 24.4 | 8.4 |
| **35-64y** | 19.3 | | 17.6 | 17.1 | 25.8 | 24.4 | 22.8 | 20.1 | 24.6 | 22.5 | 23.0 | 8.4 |
| **65+y** | 11.3 | | 25.8 | 23.8 | 17.7 | 21.5 | 15.2 | 23.2 | 23.3 | 28.6 | 25.5 | 10.9 |
| **Unspecified** | |  |  |  |  |  |  |  |  |  |  |  |
| **All persons** |  | |  |  |  |  |  |  |  |  |  |  |
| **all ages** | 44.3 | | 45.3 | 48.6 | 48.6 | 50.2 | 59.5 | 58.9 | 66.8 | 70.1 | 78.1 | 60.8 |
| **<1y** | 80.0 | | 99.2 | 69.8 | 95.8 | 83.8 | 129.0 | 125.9 | 143.7 | 91.6 | 161.8 | 127.0 |
| **1-4y** | 79.3 | | 68.7 | 90.3 | 73.5 | 78.6 | 87.4 | 96.9 | 92.5 | 115.2 | 122.1 | 116.8 |
| **5-14y** | 47.2 | | 65.2 | 65.3 | 57.8 | 68.3 | 87.9 | 76.3 | 96.6 | 93.9 | 105.4 | 79.5 |
| **15-24y** | 54.6 | | 55.8 | 56.3 | 60.6 | 65.3 | 75.5 | 77.6 | 81.9 | 96.8 | 111.4 | 84.9 |
| **25-34y** | 43.7 | | 49.6 | 49.3 | 47.2 | 50.5 | 56.5 | 57.4 | 66.1 | 69.0 | 77.9 | 53.5 |
| **35-64y** | 41.1 | | 37.2 | 43.7 | 45.2 | 42.6 | 51.1 | 53.6 | 56.4 | 60.9 | 63.1 | 51.5 |
| **65+y** | 26.3 | | 23.2 | 23.6 | 28.1 | 28.5 | 30.9 | 24.7 | 40.7 | 34.3 | 42.2 | 30.1 |
| **Males** |  | |  |  |  |  |  |  |  |  |  |  |
| **all ages** | 46.1 | | 49.2 | 48.5 | 50.8 | 51.5 | 61.1 | 60.5 | 68.7 | 70.3 | 74.7 | 59.0 |
| **<1y** | 81.0 | | 113.4 | 76.9 | 118.6 | 79.5 | 145.2 | 157.1 | 111.0 | 92.0 | 185.8 | 147.0 |
| **1-4y** | 94.5 | | 79.6 | 113.3 | 104.0 | 84.9 | 119.8 | 125.5 | 110.5 | 157.1 | 147.9 | 158.2 |
| **5-14y** | 58.2 | | 81.8 | 82.6 | 69.6 | 88.4 | 105.8 | 94.3 | 107.1 | 105.8 | 122.7 | 91.9 |
| **15-24y** | 48.5 | | 56.7 | 48.2 | 54.3 | 53.2 | 70.2 | 61.6 | 74.5 | 82.6 | 86.9 | 66.0 |
| **25-34y** | 38.7 | | 48.1 | 41.6 | 39.7 | 48.6 | 49.1 | 56.4 | 66.5 | 64.0 | 67.9 | 46.7 |
| **35-64y** | 44.1 | | 38.8 | 41.8 | 48.8 | 44.5 | 49.0 | 53.3 | 56.5 | 55.1 | 56.4 | 44.5 |
| **65+y** | 26.8 | | 27.0 | 19.9 | 24.1 | 26.3 | 31.6 | 23.5 | 47.0 | 43.2 | 42.7 | 32.6 |
| **Females** |  | |  |  |  |  |  |  |  |  |  |  |
| **all ages** | 42.5 | | 41.4 | 48.5 | 46.5 | 48.9 | 57.8 | 57.3 | 65.1 | 70.0 | 81.5 | 62.6 |
| **<1y** | 78.9 | | 84.5 | 62.3 | 71.7 | 88.4 | 112.0 | 92.6 | 178.8 | 91.1 | 136.3 | 105.8 |
| **1-4y** | 63.5 | | 57.3 | 66.3 | 41.5 | 72.1 | 53.6 | 67.0 | 73.6 | 70.7 | 94.5 | 72.5 |
| **5-14y** | 35.6 | | 48.0 | 47.4 | 45.4 | 47.3 | 69.1 | 57.6 | 85.5 | 81.3 | 87.2 | 66.4 |
| **15-24y** | 61.0 | | 54.7 | 64.8 | 67.3 | 78.1 | 81.0 | 94.6 | 89.7 | 111.9 | 137.5 | 104.9 |
| **25-34y** | 49.2 | | 51.3 | 57.4 | 55.3 | 52.6 | 64.4 | 58.4 | 65.7 | 74.1 | 87.9 | 60.3 |
| **35-64y** | 38.2 | | 35.6 | 45.7 | 41.7 | 40.7 | 53.3 | 53.9 | 56.4 | 66.8 | 69.9 | 58.5 |
| **65+y** | 26.1 | | 19.8 | 26.4 | 32.6 | 30.3 | 30.1 | 25.7 | 35.5 | 26.2 | 41.9 | 28.2 |


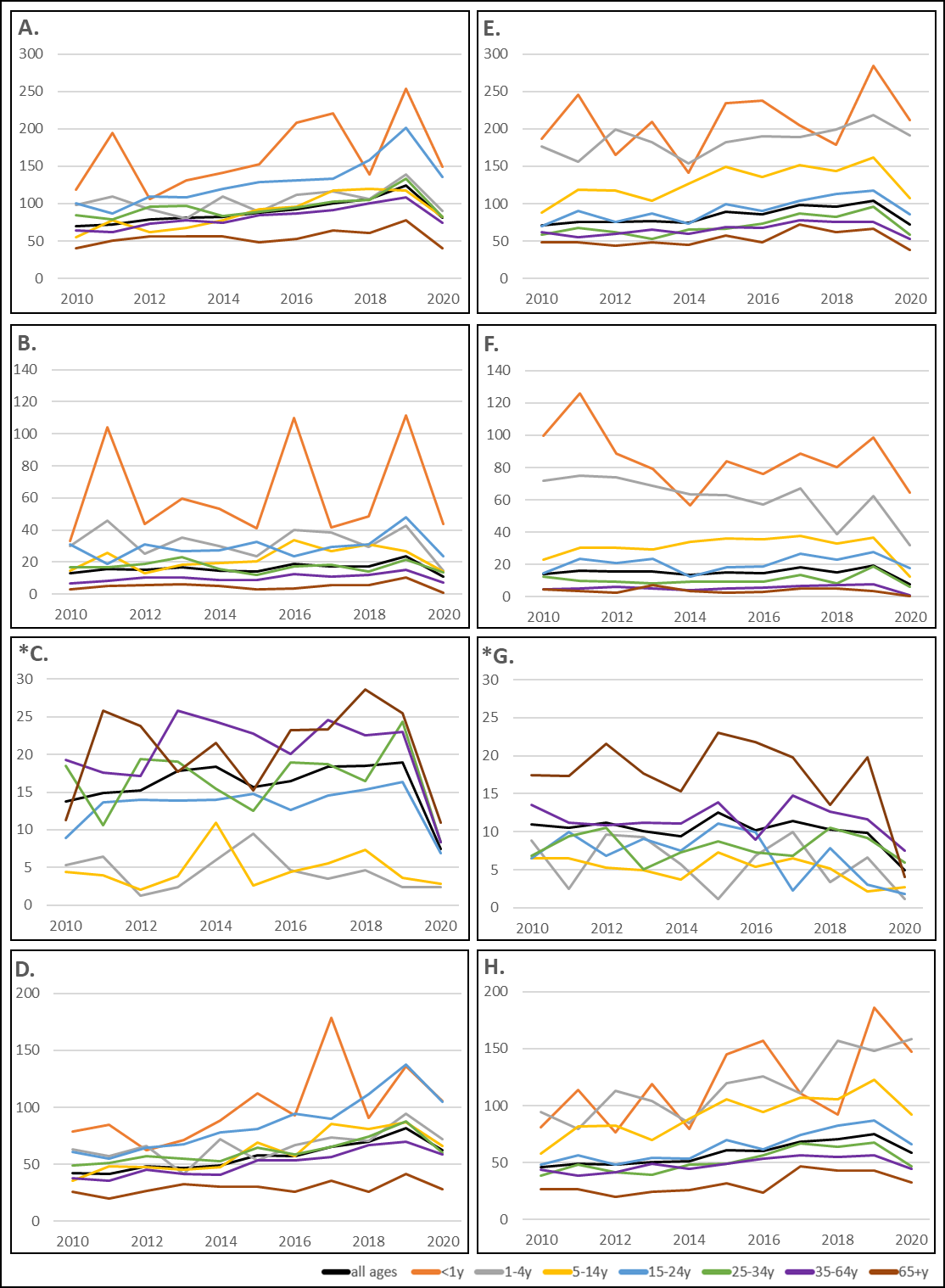


**Fig 1.** Age-standardised anaphylaxis event rates per 100,000 person-years, 2010 to 2020.

**A.** female all-cause, **B.** female food, **C.** female medication, **D.** female unspecified

**E.** male all-cause, **F.** male food, **G.** male medication, **H.** male unspecified

* Due to small numbers the <1 year and 1-4 year age groups were aggregated.

Age group 0-4y for medication:
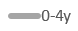

Supplement: Multimedia component 1 [file mmc1.docx]
